# Delayed diagnosis of cholangiocarcinoma presenting with shoulder pain: A case report

**DOI:** 10.1097/MD.0000000000040177

**Published:** 2024-10-25

**Authors:** Jaesuk Kim, Seongjin Park, So Young Kwon

**Affiliations:** a Department of Anesthesiology and Pain Medicine, The Catholic University of Korea, St. Vincent’s Hospital, Suwon, Republic of Korea.

**Keywords:** cholangiocarcinomas, pain, referred pain, shoulder

## Abstract

**Rationale:**

Shoulder pain is a common type of musculoskeletal pain. While musculoskeletal issues are primary causes of shoulder pain, it is important to note that referred pain in the shoulder area can also originate from non-musculoskeletal problems.

**Patient concerns:**

A 60-year-old male presented with a month-long stabbing pain in the right shoulder that was worsened by deep breathing. He had no trauma history or neurological symptoms. He also experienced a 5 kg weight loss over 3 months. Physical examination was normal. Shoulder X-ray suggested degenerative arthritis. Despite medication including opioids, his pain persisted and worsened to a 10/10 severity, spreading to the right flank and anterior chest.

**Diagnosis:**

An abdominal CT scan revealed multiple hepatic nodules, ascites, and right pleural effusion, suggesting a systemic condition.

**Interventions:**

This prompted immediate referral to oncology, where subsequent investigations confirmed the diagnosis of intrahepatic cholangiocarcinoma.

**Outcomes:**

The patient deteriorated and passed away during the buildup phase for cancer treatment.

**Lessons:**

This case underscores the importance of considering systemic conditions in patients presenting with seemingly localized symptoms such as shoulder pain. It highlights the significance of thorough evaluation and prompt referral for further investigations when necessary.

## 1. Introduction

Shoulder pain is the third most common type of musculoskeletal pain, occurring frequently in the population.^[1]^ Approximately 3% to 4% of individuals experience shoulder pain, with a significant proportion seeking medical attention.^[2]^ While musculoskeletal issues are primary causes of shoulder pain, it is important to note that referred pain in the shoulder area can also originate from non-musculoskeletal problems.^[3]^ Simple musculoskeletal shoulder pain typically originates from inflammation. It is commonly associated with motion pain.^[4]^ Conversely, shoulder pain from non-musculoskeletal origins often lacks a direct relationship with shoulder motion.

Referred pain occurs when stimuli originating from 1 part of the body are perceived as pain in another part of the body. Referred shoulder pain is believed to be primarily caused by irritation of the diaphragm. The central portion of the diaphragm is innervated by the sensory branch of the diaphragmatic nerve, which arises from cervical nerves C3 to C5. Meanwhile, the shoulder region is innervated by the sensory branch of the supraclavicular nerve also originating from the same cervical nerves, particularly C3 to C4.^[5]^ This anatomical arrangement suggests that referred shoulder pain can occur when there is irritation or pathology affecting these nerves, leading to pain perception in the shoulder region. Therefore, conditions such as gallstones or cardiac issues such as myocardial infarction can manifest as referred shoulder pain. However, gastrointestinal or hepatic problems that cause referred shoulder pain are less frequent.^[6]^ If musculoskeletal problems coexist, it can be easy to overlook referred pain or comorbidities. Missing these could lead to serious consequences for the patient.

We report a case of delayed cancer diagnosis due to the patient’s shoulder pain presenting as typical musculoskeletal pain, which initially did not suggest referred pain.

## 2. Case presentation

A 60-year-old male presented to the pain clinic with complaints of right shoulder pain, specifically involving the right scapular median border and the right middle trapezius muscle. The pain had commenced 1 month prior to the visit. It was not associated with any significant trauma or prior treatment. He described the pain as throbbing and stinging, rating it as 3 out of 10 on a numerical rating scale. The pain exacerbated to 5 out of 10 on the scale when taking deep breaths but improved when at rest. There were no accompanying neurological or neuropathic symptoms reported. Additionally, the patient had experienced an unintended weight loss of 5 kg over the past 3 months.

The patient’s past medical history included hypertension diagnosed in 2009, along with a hypertensive intracranial hemorrhage in the same year. He had been effectively managed with antihypertensive medication. He had been regularly followed up at 6-month intervals. Additionally, 1 week prior to the visit to the pain clinic, he was diagnosed with uncontrolled diabetes and initiated insulin therapy. There were no significant familial medical histories reported.

Upon inspection, no abnormalities were observed. A comprehensive physical examination was conducted. The range of motion of the right shoulder was assessed, revealing forward flexion of 130°, abduction of 100°, external rotation of 70°, and internal rotation reaching the T10 level. The patient reported pain at approximately 50° of abduction. Both the Neer test and Hawkin test elicited positive findings at the right shoulder. Examination of the cervical spine movement revealed no abnormalities. Its ROM was within normal limits. Furthermore, there was no exacerbation of symptoms noted during the Spurling test.

**Figure 1.**
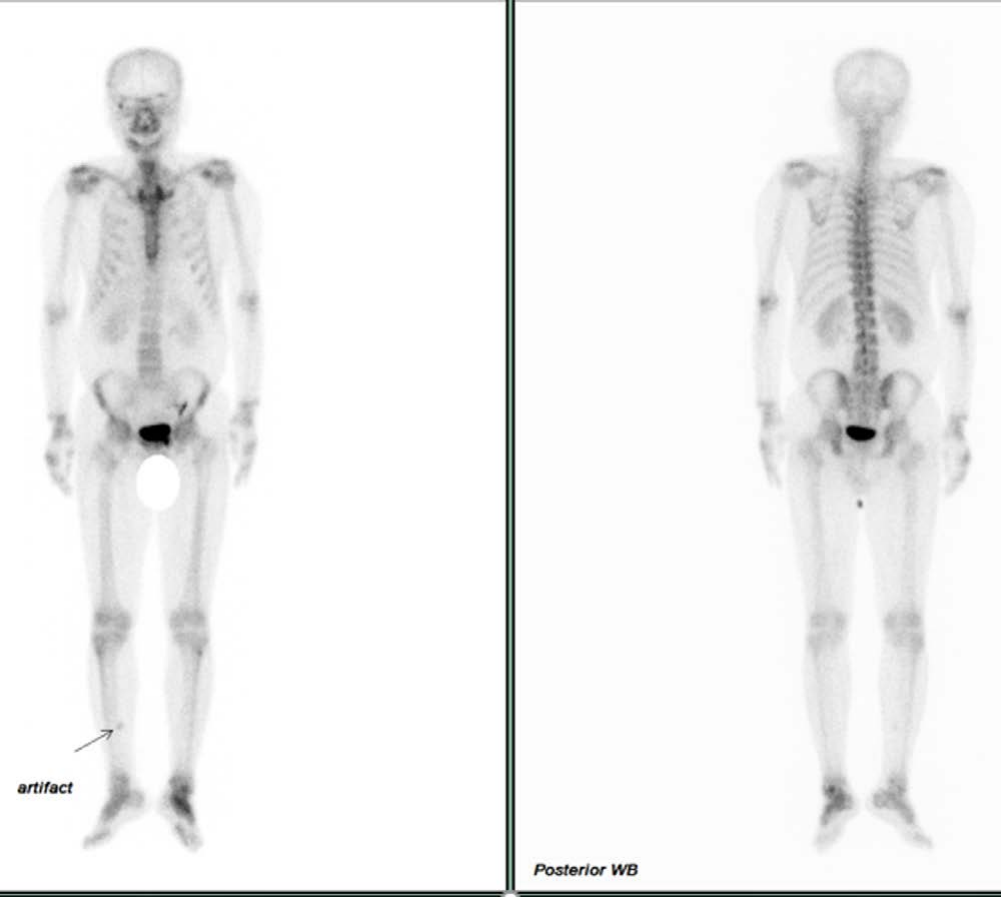
Bone scan showing arthritis of bilateral shoulders, wrists, fingers, knees, ankles, and feet.

Following further evaluation, including right shoulder X-ray, cervical spine X-ray, and bone scan examinations, several findings were noted. The right shoulder X-ray revealed an ovoid sclerotic lesion at the right humeral head. The cervical spine X-ray indicated a bulging disc and minimal hypertrophic uncovertebral joint changes at the C5 to C6 level, along with minimal right foraminal stenosis at the C4 to C5 level. The bone scan revealed degenerative spondylosis in the lumbar vertebrae and arthritis affecting bilateral shoulders, wrists, fingers, knees, ankles, and feet (Fig. 1).

Based on these findings, the initial impression was degenerative arthritis of the right shoulder. Treatment was initiated with oral medications, including gabapentin 200 mg twice daily, Afloqualone 80 mg twice daily, and Mypol capsules twice daily.

Following the initial treatment, the patient reported mild pain relief of approximately 10% to 20% for 2 days. However, the pain worsened thereafter, reaching a severity of 7 out of 10 on a numerical rating scale. To manage the escalating pain, the patient commenced opioid therapy, including Targin 20 mg twice daily and hydromorphone 4.36 mg once daily for 1 week.

Given the lack of significant improvement in pain despite opioid therapy, an MRI of the right shoulder was conducted. The MRI revealed a tear in the supraspinatus tendon, along with a curved acromion and a traction-type subacromial spur.

A week later, the patient reported that the pain had intensified to 10 out of 10 on a numerical rating scale, extending to his right flank and right anterior chest. Recognizing the need to rule out underlying medical conditions, the patient was promptly evaluated through the emergency room.

**Figure 2.**
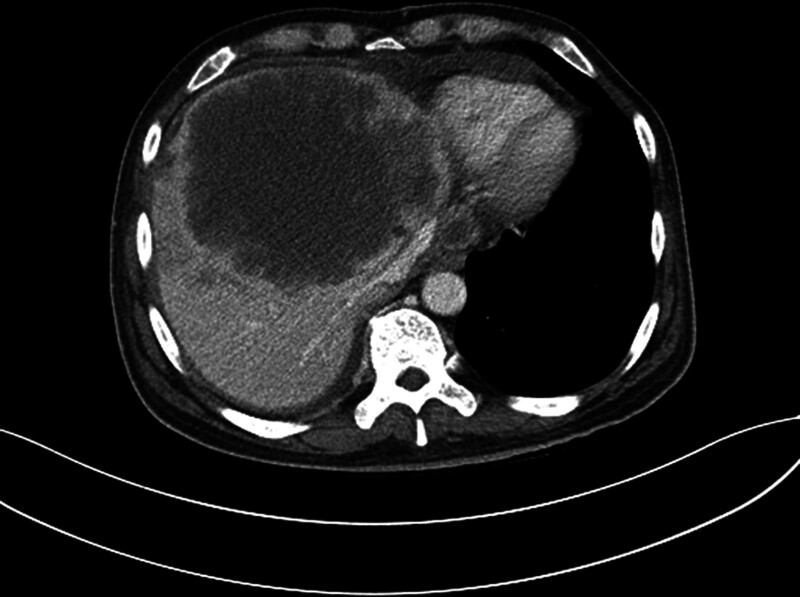
Contrast-enhanced abdominal CT revealing ill-defined heterogenous enhancing hepatic mass more than 12 cm × 11 cm.

An abdomen CT scan revealed a heterogeneous enhancing hepatic mass measuring 12 cm × 11 cm at the S8/S4 segment, along with multiple hepatic nodules and the presence of ascites and right lower pleural effusion (Fig. 2). Subsequently, the patient was transferred to the oncology department, where a diagnosis of intrahepatic cholangiocarcinoma with hepatic metastasis was confirmed. A chemotherapy regimen was planned. However, the patient deteriorated and passed away during the buildup phase for cancer treatment.

## 3. Discussion

In this case, there are 2 plausible explanations for the delayed diagnosis.

Firstly, the rarity of cholangiocarcinoma itself contributes to its challenging diagnosis.^[7]^ With an incidence of only about 2%, cholangiocarcinoma is not readily considered as a differential diagnosis.^[8]^ Moreover, cholangiocarcinoma is notorious for its lack of distinctive clinical symptoms, with presentations varying significantly based on tumor location and size.^[9]^ This variability complicates early suspicion. Typically, patients with cholangiocarcinoma are diagnosed after the cancer has advanced to some degree.^[9]^ Particularly in cases of intrahepatic cholangiocarcinoma, like in this instance, patients often exhibit nonspecific symptoms such as abdominal pain, malaise, night sweats, and cachexia.^[10]^ However, during the initial visit to the pain clinic, the patient solely complained of right shoulder pain without accompanying symptoms such as abdominal pain or night sweats. This absence of additional symptoms made malignancy a challenging consideration.

The second reason for the delayed diagnosis is the presence of underlying conditions that could potentially cause shoulder pain. Shoulder pain can stem from various factors, including rotator cuff disorders, glenohumeral disorders, acromioclavicular joint disease, and referred neck pain.^[11]^ In the present case, the patient presented with typical musculoskeletal shoulder pain. Consequently, we performed a bone scan and X-ray to identify any abnormalities in the bone and muscle. Initial imaging studies showed a sclerotic lesion on the humeral head on shoulder X-ray with findings of foraminal stenosis on cervical spine X-ray. Despite the absence of abnormalities in range of motion and other physical examination findings, these abnormal imaging results prompted suspicion of shoulder pathology rather than pain referred from systemic conditions. Moreover, the subsequent shoulder MRI confirmed the presence of a rotator cuff tear, further directing attention towards localized shoulder issues and potentially overshadowing considerations of referred pain from systemic conditions. This emphasis on localized pathology might contribute to the oversight of underlying systemic conditions such as intrahepatic cholangiocarcinoma ultimately diagnosed in this case.

In this case, the right shoulder pain could also be explained by Kehr sign, which suggests referred pain from diaphragmatic irritation. The lesion located near the liver likely contributed to the diaphragmatic irritation, leading to referred pain in the right shoulder. Kehr sign is typically associated with lesions affecting the right side of the diaphragm, such as liver pathologies, while left shoulder pain may indicate issues related to the spleen or stomach.^[12]^ This highlights the importance of considering Kehr sign when patients present with atypical symptoms of referred shoulder pain. This discussion underscores the significance of recognizing non-musculoskeletal sources of pain to prevent misdiagnosis or delayed diagnosis, particularly when systemic symptoms such as weight loss are present.

Although mechanisms of referred pain have not been fully elucidated yet, a complex interplay of nociceptive and neuropathic pain often leads to diagnostic challenges.^[13]^ It is important to recognize that various disorders, including those unrelated to the musculoskeletal system, can be masked within referred pain.^[3]^ Therefore, thorough assessment, including the identification of Red Flags, is crucial.^[14]^ While not definitive for diagnosis, Red Flags can raise clinical suspicion.^[15]^ In cases of shoulder pain, Red Flags suggestive of underlying abdominal pathology may include symptoms such as abdominal pain, hypochondriac pain, and epigastric pain.^[6]^ Additionally, the presence of systemic symptoms such as weight loss, generalized joint pain, fever, lymphadenopathy, and new respiratory symptoms should prompt consideration of non-musculoskeletal diseases. These Red Flags provide important cues to guide further evaluation and ensure comprehensive patient care.^[11]^

The unintentional weight loss of 5 kg over a span of 3 months should have raised suspicion as a significant Red Flag. Weight loss, especially when not attributed to intentional efforts, can often be indicative of underlying systemic issues including malignancy. In the present case, if weight loss had been recognized as a Red Flag and investigated more thoroughly, it might have prompted a comprehensive systemic examination, potentially leading to an earlier diagnosis.

This case highlights the importance of considering the possibility of referred pain from conditions such as cancer when a patient presents with typical musculoskeletal shoulder pain and performing examinations such as X-ray and bone scan. Particularly in the presence of red flag signs, it is crucial to conducting a thorough evaluation to avoid missing subtle hints such as unintentional weight loss. By being more attuned to such indicators and conducting comprehensive assessments, clinicians can facilitate earlier detection and intervention, leading to improved patient outcomes.

## 4. Conclusion

In primary care settings, where patients with shoulder pain are initially assessed, it is imperative to move beyond routine shoulder range of motion and shoulder-specific tests. Instead, clinicians should adopt a comprehensive approach, which involves taking a detailed medical history, conducting a thorough physical examination, and being vigilant for any Red Flags suggestive of systemic pathology. By doing so, clinicians can ensure that potential underlying conditions are not overlooked, facilitating earlier diagnosis and timely intervention. This proactive approach ultimately leads to better patient outcomes. It could prevent potential complications associated with delayed diagnosis.

## Author contributions

**Conceptualization:** So Young Kwon.

**Investigation:** Jaesuk Kim, Seongjin Park, So Young Kwon.

**Project administration:** So Young Kwon.

**Resources:** So Young Kwon.

**Writing – original draft:** Jaesuk Kim.

**Writing – review & editing:** Jaesuk Kim, Seongjin Park, So Young Kwon.

## References

[1] Gallardo Vidal MI Calleja Delgado L Tenezaca Marcatoma JC Calleja Guadix I Daimiel Yllera A Morales Tejera D. [Physiotherapy and health education protocol in chronic musculoskeletal shoulder pain. Experience in Primary Care]. Aten Primaria. 2022;54:102284. 35461039 10.1016/j.aprim.2022.102284 PMC9046942

[2] Lucas J van Doorn P Hegedus E Lewis J van der Windt D. A systematic review of the global prevalence and incidence of shoulder pain. BMC Musculoskelet Disord. 2022;23:1073. 36476476 10.1186/s12891-022-05973-8 PMC9730650

[3] Lollino N Brunocilla PR Poglio F Vannini E Lollino S Lancia M. Non-orthopaedic causes of shoulder pain: what the shoulder expert must remember. Musculoskelet Surg. 2012;96(Suppl 1):S63–8. 22528847 10.1007/s12306-012-0192-5

[4] Dieppe PA Lohmander LS. Pathogenesis and management of pain in osteoarthritis. Lancet. 2005;365:965–73. 15766999 10.1016/S0140-6736(05)71086-2

[5] Keith L. Moore AFD Anne M Agu R. Clinically Oriented Anatomy. 8th ed: Lippincott Williams & Wilkins; 2017:671–819.

[6] Pennella D Giagio S Maselli F. Red flags useful to screen for gastrointestinal and hepatic diseases in patients with shoulder pain: a scoping review. Musculoskeletal Care. 2022;20:721–30. 35229444 10.1002/msc.1628

[7] Khan AS Dageforde LA. Cholangiocarcinoma. Surg Clin North Am. 2019;99:315–35. 30846037 10.1016/j.suc.2018.12.004

[8] Kawamura R Harada Y Shimizu T. Missed diagnosis of cholangiocarcinoma presenting with atypical symptoms. Eur J Case Rep Intern Med. 2021;8:002207. 33585340 10.12890/2021_002207 PMC7875580

[9] Blechacz B Komuta M Roskams T Gores GJ. Clinical diagnosis and staging of cholangiocarcinoma. Nat Rev Gastroenterol Hepatol. 2011;8:512–22. 21808282 10.1038/nrgastro.2011.131 PMC3331791

[10] El Rassi ZE Partensky C Scoazec JY Henry L Lombard-Bohas C Maddern G. Peripheral cholangiocarcinoma: presentation, diagnosis, pathology and management. Eur J Surg Oncol. 1999;25:375–80. 10419707 10.1053/ejso.1999.0660

[11] Mitchell C Adebajo A Hay E Carr A. Shoulder pain: diagnosis and management in primary care. Bmj. 2005;331:1124–8. 16282408 10.1136/bmj.331.7525.1124 PMC1283277

[12] Söyüncü S Bektaş F Cete Y. Traditional Kehr’s sign: left shoulder pain related to splenic abscess. Ulus Travma Acil Cerrahi Derg. 2012;18:87–8. 22290058 10.5505/tjtes.2011.04874

[13] Russo MM Sundaramurthi T. An overview of cancer pain: epidemiology and pathophysiology. Semin Oncol Nurs. 2019;35:223–8. 31085106 10.1016/j.soncn.2019.04.002

[14] Maselli F Rossettini G Viceconti A Testa M. Importance of screening in physical therapy: vertebral fracture of thoracolumbar junction in a recreational runner. BMJ Case Rep. 2019;12:e229987. 10.1136/bcr-2019-229987 PMC6733320 31471360

[15] Maselli F Palladino M Barbari V Storari L Rossettini G Testa M. The diagnostic value of Red Flags in thoracolumbar pain: a systematic review. Disabil Rehabil. 2022;44:1190–206. 32813559 10.1080/09638288.2020.1804626

